# The *CMJ* stays in the editor’s heart 

**DOI:** 10.3325/cmj.2026.67.53

**Published:** 2026-04

**Authors:** Svjetlana Kalanj Bognar

**Affiliations:** *Croatian Medical Journal*, Zagreb, Croatia

My journey as the editor-in-chief of the *Croatian Medical Journal* (*CMJ*) began in the spring of 2019. The first editorial I wrote in this capacity reflected on the continuity through changes of the journal’s policy and management (1). And changes are, of course, necessary to maintain the quality of the journal’s contents, visibility, and recognition within the international scientific community. However, from time to time, changes mean introducing a new editorial team. With this in mind, this editorial announces a change in the *CMJ* editorial board and my farewell from the journal’s leading position after two mandates.

The editorial role imprinted tremendously on my professional and personal life, and while entering a new cycle of academic duties inevitably leads to my stepping down as the editor-in-chief, I am grateful and proud for having been a part of more than 30 years of the journal’s story. The journal was born back in the 1990s, when its first editors-in-chief strove to create a serious and professional medical journal in the middle of a war, in a country pinned at the margins of the scientific world map. This bold idea has meanwhile materialized with exceptional success. A small academic journal has found its place in the publishing arena, earning respect, reputation, and recognition by all the relevant bibliographic databases in the field. Yes, there have been many obstacles on this path; however, the enthusiasm of the journal’s editors and contributors has managed to surpass them. The *CMJ* sails on with confidence and caution, in spite of the unforeseen global events occurring since 2019, all of them affecting the scientific publishing. In less than a decade, the world has encountered a pandemic, accelerated climate change, and escalation of conflicts. The pandemic disturbed health care systems, dismantling many of their negative aspects, and incited anti-science movements, but also led to a fast discovery and implementation of an efficient vaccine. Scientific publishing has been shaken up as well, with massive paper production influencing the bibliometric analytics. Many new journals have emerged with surprisingly high rankings and impact factors, while many old journals ceased to exist or lost their reputation, not necessarily due to their scientific quality. As if this has not been challenging enough, new questions and worries are already here: cyber-attacks aimed at impersonating official journal websites, mostly targeting small, non-profitable journals, and exponential rise of artificial intelligence use and misuse in publishing. The *CMJ* has been witnessing and commenting on all these events (2-5). Additionally, a strong earthquake hit Zagreb in 2020, just a month after the pandemic was declared, damaging the premises of the Zagreb University School of Medicine where the editorial office is situated.

Nevertheless, the journal has been running continuously thanks to efforts of the entire editorial team, as is clearly reflected in the following numbers and facts. Since 2019, we have published 554 articles that aggregated 2799 citations so far (according to the Web of Science). We have managed more than 500 manuscripts submitted per year (desk rejection rate of around 85%), publishing 6 issues per year, engaging expert and statistical reviewers, performing language editing of each word and sentence, preparing with precision graphical and technical material, and enhancing our visual identity with attractive artistic covers. Importantly, the journal stands solid in bibliometric terms – our impact factor has risen from 1.247 (2019) to 2.3 (released last year for 2024 by Clarivate), and the journal was ranked in Q2 in 2023 and 2024. Even beyond the impact factor (6), the *CMJ* certainly exerts significant educational and regional impact.

And for me, the impact of working in the journal has been immeasurable – intellectually, academically, and emotionally. It has been demanding, easy, funny, worrisome, and intriguing at the same time. I have gained new friends and colleagues, and probably lost some of them (hopefully not forever). And it has left in me the feeling of being home, with my dearest ones, with everything I love or do not love about them. I am sure that I am not alone in this feeling, and that the *CMJ* stays permanently in the hearts of all its editors. With deepest gratitude to all my associates for being beside me in both challenging and peaceful moments, I remain certain that the *CMJ* will continue its successful, productive, and respectable publishing journey in the hands of a new captain and his crew!
